# Supplementary material for: Guettarda crispiflora Vahl Methanol Extract Ameliorates Acute Lung Injury and Gastritis by Suppressing Src Phosphorylation
Source: Plants (Basel). 2022 Dec 16;11(24):3560. doi: 10.3390/plants11243560 (PMC9784507; doi:10.3390/plants11243560)
Supplement: Supplementary file 1 [file plants-11-03560-s001.zip › plants-2047523-supplementary.pdf]

Supplementary materials

Guettarda crispiflora Vahl Methanol Extract Ameliorates Acute Lung Injury and Gastritis by Suppressing Src Phosphorylation

Dahae Lee [1,†], Ji Won Kim [1,†], Chae Young Lee [1], Jieun Oh [1], So Hyun Hwang [1], Minkyeong Jo [1], Seung A Kim [1], Wooram Choi [1], Jin Kyoung Noh [2], Dong-Keun Yi [3], Minkyung Song [1,*], Han Gyung Kim [1,4,5,*] and Jae Youl Cho [1,4,5,*]

1 Department of Integrative Biotechnology, Sungkyunkwan University, Suwon 16419, Republic of Korea
2 Instituto de BioEconomia, Quito 170135, Ecuador
3 International Biological Material Research Center, Korea Research Institute of Bioscience and Biotechnology, Daejeon 34141, Republic of Korea
4 Research Institute of Biomolecule Control, Sungkyunkwan University, Suwon 16419, Republic of Korea
5 Biomedical Institute for Convergence at SKKU, Sungkyunkwan University, Suwon 16419, Republic of Korea
* Correspondence: piscesmk@skku.edu (M.S.); hanks523@skku.edu (H.G.K.); jaecho@skku.edu (J.Y.C.)
† These authors equally contributed to this work.

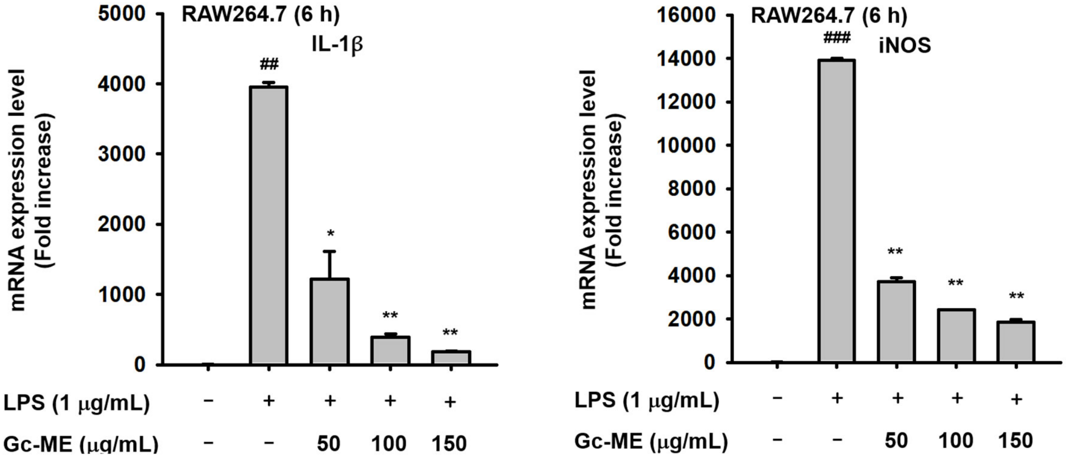

(A) (B)

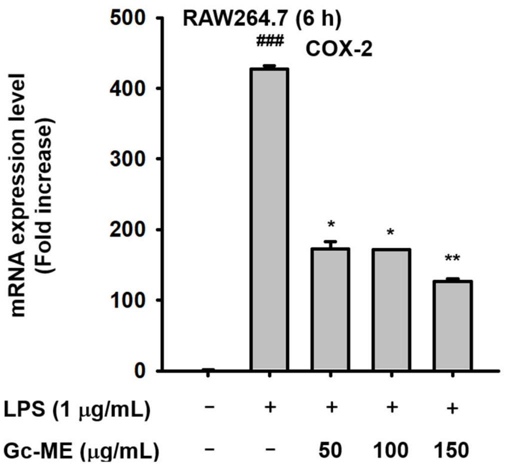

(C)

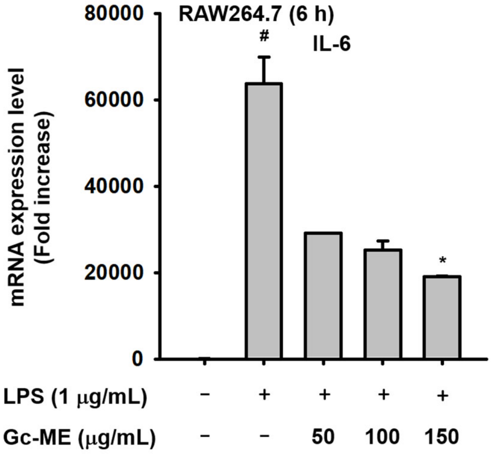

(D)

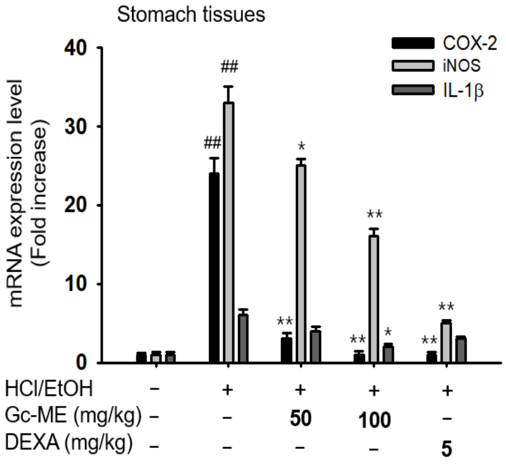

(E)

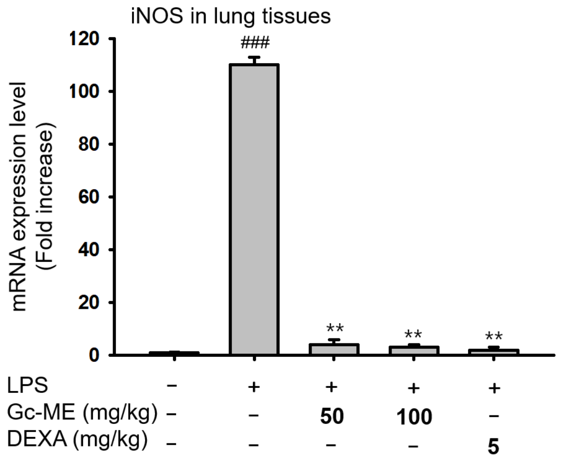

(F)

Figure S1. The inhibitory effect of Gc-ME on the expression of pro-inflammatory genes. (A-F) mRNA levels of pro-inflammatory genes (IL-1β, iNOS, COX-2 and IL-6) were measured by real-time PCR (qRT-PCR) with RNA samples prepared from LPS-treated RAW264.7 cells pretreated with Gc-ME for 30 min (A-D) or tissue (stomach and lung) lysates from Gc-ME-administered mice treated with HCl/EtOH or LPS (E and F). Results (A–F) are expressed as mean ± standard deviation. #: $p < 0.05$, ###: $p < 0.01$, and ###: $p < 0.001$ compared to control group (no treatment); *: $p < 0.05$ and **: $p < 0.01$ compared to control group (LPS alone) by Student's *t*-test; −, no treatment; +, treatment.

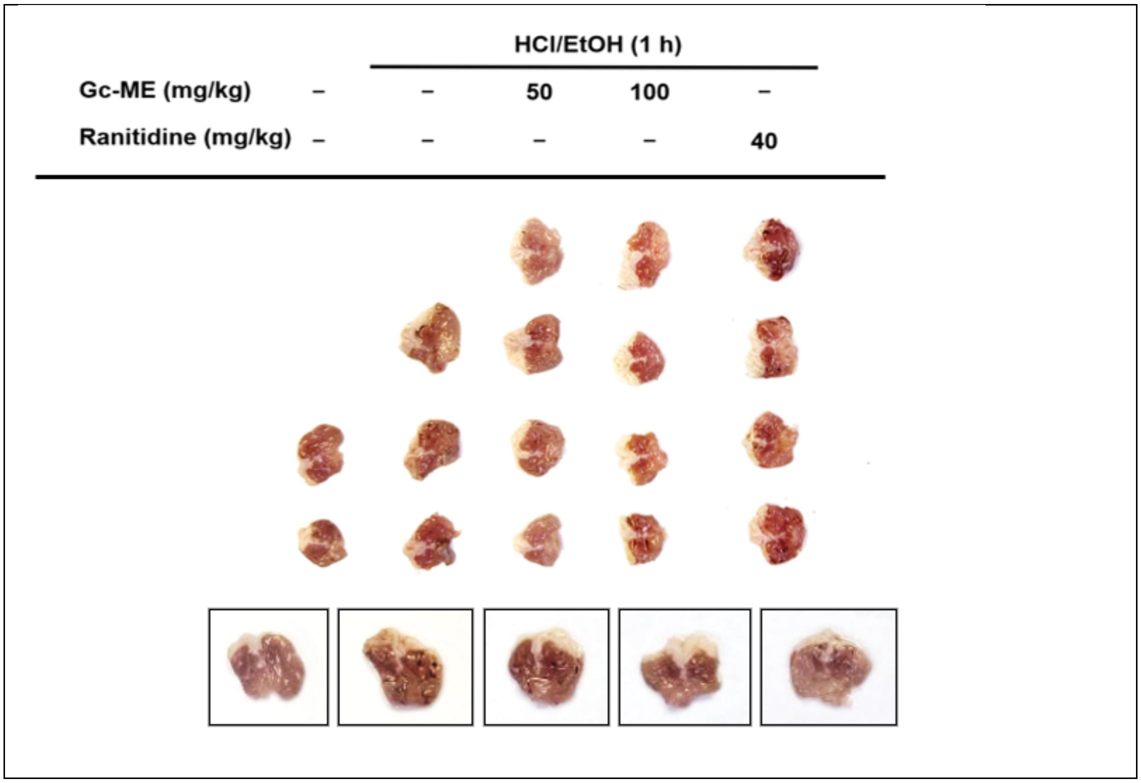

Figure S2. The inhibitory effect of Gc-ME on the induction of gastric lesion in stomach of mice treated with HCl/EtOH and Gc-ME. Photos of stomachs were taken with a digital camera.
